# Derivation of Hair-Inducing Cell from Human Pluripotent Stem Cells

**DOI:** 10.1371/journal.pone.0116892

**Published:** 2015-01-21

**Authors:** Ksenia Gnedeva, Ekaterina Vorotelyak, Flavio Cimadamore, Giulio Cattarossi, Elena Giusto, Vasiliy V. Terskikh, Alexey V. Terskikh

**Affiliations:** 1 Department of Developmental and Stem Cell Biology, Sanford-Burnham Medical Research Institute, La Jolla, CA, United States of America; 2 Laboratory of Cell Proliferation, Koltsov Institute of Developmental Biology, Moscow, Russia; Baylor College of Medicine, UNITED STATES

## Abstract

Dermal Papillae (DP) is a unique population of mesenchymal cells that was shown to regulate hair follicle formation and growth cycle. During development most DP cells are derived from mesoderm, however, functionally equivalent DP cells of cephalic hairs originate from Neural Crest (NC). Here we directed human embryonic stem cells (hESCs) to generate first NC cells and then hair-inducing DP-like cells in culture. We showed that hESC-derived DP-like cells (hESC-DPs) express markers typically found in adult human DP cells (e.g. p-75, nestin, versican, SMA, alkaline phosphatase) and are able to induce hair follicle formation when transplanted under the skin of immunodeficient NUDE mice. Engineered to express GFP, hESC-derived DP-like cells incorporate into DP of newly formed hair follicles and express appropriate markers. We demonstrated that BMP signaling is critical for hESC-DP derivation since BMP inhibitor dorsomorphin completely eliminated hair-inducing activity from hESC-DP cultures. DP cells were proposed as the cell-based treatment for hair loss diseases. Unfortunately human DP cells are not suitable for this purpose because they cannot be obtained in necessary amounts and rapidly loose their ability to induce hair follicle formation when cultured. In this context derivation of functional hESC-DP cells capable of inducing a robust hair growth for the first time shown here can become an important finding for the biomedical science.

## Introduction

It has long been suggested that in embryogenesis hair follicles are formed by reciprocal interactions between the epidermis and underlying mesoderm [1,2,3,4]. Dermal Papillae (DP) first arise as cell condensates in the dermis in response to epidermal placode formation. As hair follicles progress in development, epidermal cells in placodes proliferate actively and envelope the dermal condensates, now called dermal papillae, separating them from surrounding dermis [5]. Exposed to these new niche conditions, DP cells acquire the expression of BMP-4, its inhibitor noggin, and the surface markers N-CAM and p-75. Additionally, they secret specific extracellular matrix proteins (e.g. versican (VCAN)) and show high level of alkaline phosphatase activity (AP) [6]. Using double reporter Lef1-RFP / K14-H2BGFP mice, more recent studies identified detailed genetic signature of prospectively isolated mouse DP cells [7] and identified Wnt, BMP and FGF singling pathway as key requirement for murine DP maintenance and function [8,9,10]. DP cells play a critical role in hair growth and cycling [6] and determine hair size and hair type [11,12]. It has been long recognized that DP cells are able to induce hair follicle formation not only in embryogenesis but also postnatal. Vibrissae DP cells induced de novo hair formation when transplanted into the footpad of the adult rat, which is normally a non-haired skin area [13]. Human DP cells isolated from scalp skin contribute to hair formation when transplanted into rodents [14,15] and induce keratinocytes morphogenesis in cultures [16]. However, extensive amplification of DP cells in culture is not feasible as they quickly lose the hair-inducing potential with passaging [7,8,17,18]. This represents a practical roadblock for the use of prospectively isolated human DP cells to develop a cell-based treatment for hair loss diseases.

Neural crest (NC) is a cell population that transiently arises from the dorsal neural tube in development and gives rise to multiple tissues including the peripheral neural system, adrenal medulla, melanocytes and various craniofacial mesenchymal tissues [19]. NC-specific (Wnt1-Cre) lineage tracing using Lox-STOP-Rosa26 or Z/EG reporter mice provided the genetic evidence of NC contribution to a large proportion of cephalic DP cells [20,21,22]. Human embryonic stem cells (hESCs) have been directed to various cell fates including hair follicle epidermal cells—keratinocytes [23], however, the derivation of DP cells have not been reported. Here we describe for the first time the derivation of functional DP-like cells from human embryonic stem cells.

## Results

### Derivation of hESC-DP using NC cells intermediate

**Figure 1:**
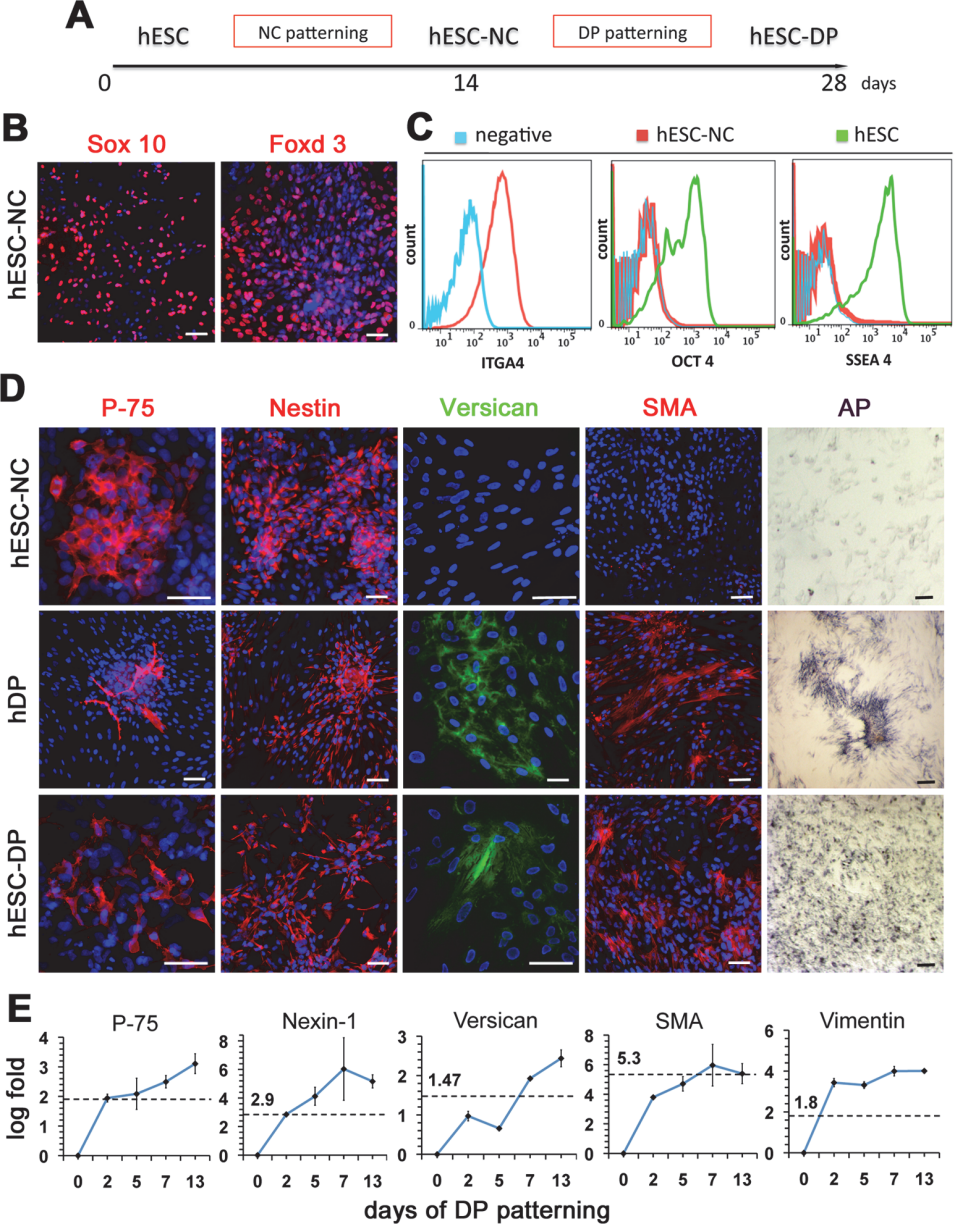
Differentiation of hESCs into DP-like cells via NC intermediate. (A) Schematic representation of the differentiation strategy. (B) Expression of migratory NC markers Sox 10 and Foxd3, in hESC-NC cultures. Immunofluorescent staining, DAPI in blue. (C) Flow cytometry analysis of NC marker integrin alpha-4 (ITGA4) and hESC markers OCT4 and SSEA4 in hESC-NC cultures. (D) Expression of p-75, Nestin, Versican, SMA and Alkaline Phosphatase (AP) in hESC-NC, hDP (from normal skin) and hESC-DP cultures. Immunofluorescent staining, DAPI in blue. (E) Q-PCR analysis of hDP markers p-75, Nexin-1, Versican, SMA and Vimentin in hESC-DP cultures during DP patterning. Day 0 = hESC-NC cells. Levels of gene expression, shown as log of fold change over hESC-NC levels, were normalized to 18S. For each gene the dashed line represents levels of gene expression in hDP cell cultures. Scale bars 100 μm.

Since the prior genetic evidence of NC contribution to DP cells in vivo [21,22], we thought to obtain human DP-like cells from human ESC via the NC intermediate (Fig. 1A). Previously we have described efficient differentiation of human ESC into the multipotent NC cells [24,25]. Identical protocol was used here to generate human ESC-derived Neural Crest cells (hESC-NC). hESC-NC cultures showed robust expression of the neural crest markers Sox10 and Foxd3 (Fig. 1B). Flow cytometry analysis confirmed that nearly 80% of cultured hESC-NC cells express the NC marker Integrin alpha 4 (ITGA4), the cognate receptor for fibronectin [26], and lack the expression of OCT4 and SSEA4 suggesting the absence of undifferentiated hESCs (Fig. 1C).

Neural crest is a multipotent population of cells that give rise to precursors for various mesenchymal tissues [19]. The FACS analysis showed that hESC-NC cells on 14 days of differentiation expressed mesenchymal stem cell markers CD47 (99.95%), CD184 (20%), and CD44 (52.58%) (S1 Fig.). To generate DP-like cells, we further differentiated hESC-NCs in DMEM-F12 medium containing 10% FBS for two additional weeks (Fig. 1A). It has been shown that DP cells are somatic dermal stem cells [27] and that they also express mesenchymal stem cell (MSC) markers [28]. Therefore, we enriched differentiating hESC-NCs cultures for mesenchymal cells using preferential adherence to tissue culture plastic, a known property of MSC [29]. Routinely, about 20% of hESC-NC cultures adhered to plastic and were passaged in serum containing media giving rise to hESC-derived DP-like cells (hESC-DP). These results suggest hESC-derived NC cells contain the mesenchymal progenitor population of cells that can be enriched using isolation protocol and culture conditions for MSC and DP cells.

### Characterization of hESC-DP cells

The signature genes of mouse dorsal skin DP cells have been compiled [7], however very little is know about the gene expression in human cephalic dermal papillae cells. In order to characterize mesenchymal hESC-DP we used the markers shown to be present in both mouse and human DP cells. In agreement with previously reported data [21], expression of the common for DP and neural crest markers p-75 and Nestin was detected in hESC-NC cells, cultured human DP cells (hDP) (isolated from normal human skin) and hESC-DP cells (Fig. 1D). The expression of the three other well established human DP markers Versican, Smooth Muscle Actin (SMA) and Alkaline Phosphatase (AP) was undetectable in hESC-NC cells but was present in hDP and hESC-DP cultures (Fig. 1D). The specificity of staining for Nestin, Versican, SMA and AP in hDP was confirmed using human scalp skin sections (S2 Fig.). The expression of NC markers P-75 (~30%) and Nestin (~90%) in hESC-NC was similar to that in hESC-DP cultures (p-75 ~40%, Nestin ~90%) and showed a close pattern of expression in hDP cells (P-75 ~20%, Nestin ~90%). In contrast, hESC-NC cells were negative for Versican (<1%), SMA (<3%) and completely lacking AP activity, whereas the majority of hESC-DP expressed Versican (~70%), SMA (~70%) and showed high level of AP activity similar to that found in human DP cells, which were nearly 100% positive for Versican, SMA and AP. The overall cell morphology and sub-cellular localization of markers were similar between the cultures of human DP cells and hESC-DPs. To quantitatively evaluate the expression dynamic of DP markers during differentiation of hESC-NC into hESC-DP we used Q-PCR analysis. We found that the expression levels of all tested human DP markers tested (p-75, Nexin-1, Versican, SMA, and Vimentin) progressively increased during hESC-NC differentiation and after two weeks were comparable or higher then that found in human-DP cell cultures (Fig. 1E). Taken together, these results suggest the presence of human DP-like cells within hESC-DP cultures.

### Hair-inducing properties of hESC-DP upon transplantation

**Figure 2:**
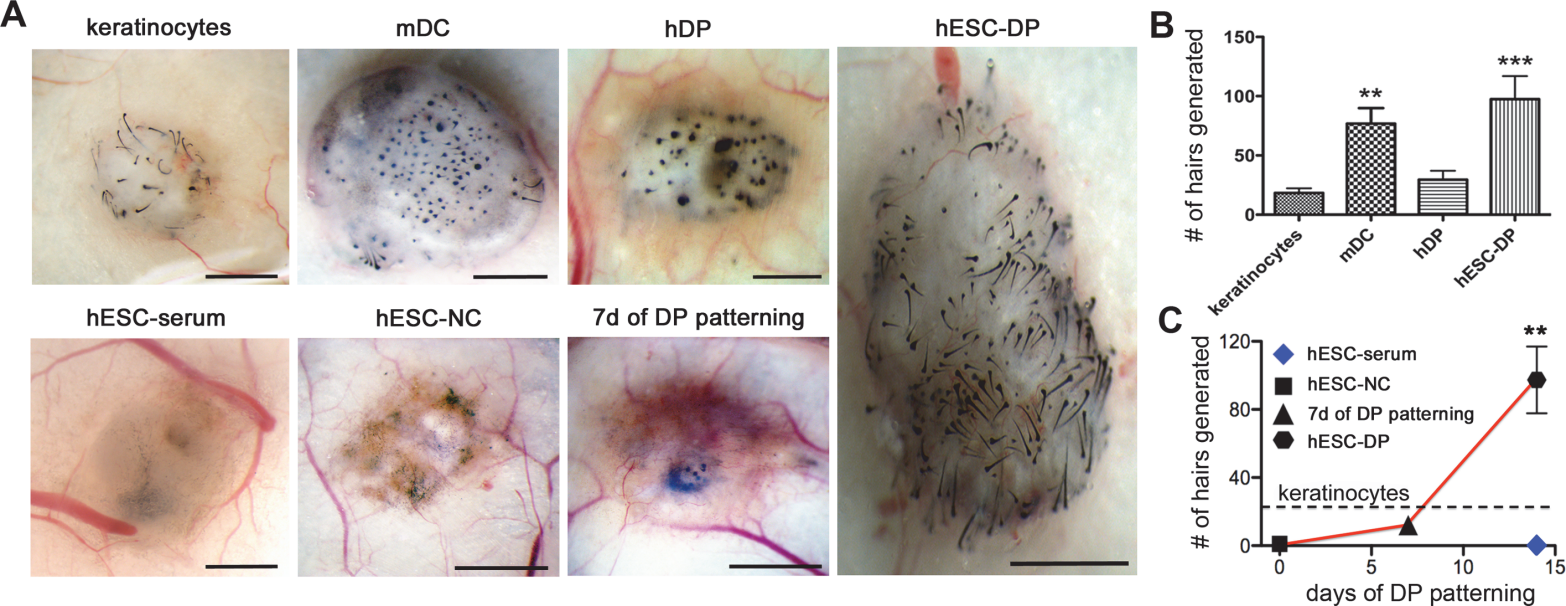
Subcutaneous cell transplantations into Nude mice. (A) Stereo images of the whole mounts of keratinocytes transplanted alone or in combination with mouse neonatal Dermal Cells (mDC), hDP, hESC-DP, hESC differentiated in serum for 14 days (hESC-serum), human hESC-derived Neural Crest cells (hESC-NC) and hESC-NC differentiated to DP for 7 days (hESC-DP 7 days). (B) Quantification hairs induced by keratinocytes transplanted alone or in combination with mDC, hDP or hESC-DP. (C) Dynamics of hair inductive capability of ESC-DP cells with time of differentiation from hESC-NC (day 0) shown as number of hairs formed per transplantation (trend visualized by the red line) or hESC differentiated in presence of serum (blue diamond) in comparison with keratinocytes alone (visualized by the dashed line). All data are represented as mean ± SEM and were analyzed with one-way ANOVA (Kruskal-Wallis test, Dunn’s Multiple Comparison post test). *, P ≤ 0.05; **, P ≤ 0,001. Scale bars 1 mm.

Next, we investigated whether hESC-DP cells are competent to induce the formation of hair follicles upon transplantation in athymic nu/nu (Nude) mice. We took advantage of the patch method of cell transplantation previously used to demonstrate the hair-inducing potential of mouse skin-derived dermal precursors [27]. Briefly, cells of interest were combined with mouse epidermal cells (keratinocytes) isolated from the newborn animals and transplanted subcutaneously into the Nude mice as a thick cell suspension. Because Nude mice have the BALB/c (albino) genetic background, we were able to distinguish between newly formed and preexisting hairs by using the epidermal cells from dark haired C57BL/6 mice for transplantation. Hair-inducing capacity was measured as the number of hairs formed per transplant. Patch method does not allow newly formed hairs to enter the skin surface that perturbs hair follicle morphology on the advanced stages of morphogenesis. Therefore the analysis was carried out at 14 days post transplantation when hair follicles were formed, but not fully developed. Consistent with the previously published experiments, transplantation of epidermal cells alone resulted in minimal hair induction, likely due to the presence of endogenous DP cells (Fig. 2A, 2B). The mouse dermal cells (mDC), used as the positive control, induced robust hair growth (P = 0.0282) with efficiency similar to that reported previously for same transplantation model [27](Fig. 2A, 2B). As expected, cultured human DP cells isolated from adult scalp skin didn’t induce a significant number of hairs compare to the negative control (keratinocytes alone) (Fig. 2A, 2B). Indeed, human DP cells have been shown to contribute in trans-species reformation of single hairs [14] but the robust hair-inducing capability of human DP cells in the mouse model has not been reported [18]. In contrast, we observed significant (P = 0.0002) hair-induction by hESC-DPs similar to that of mDC (Fig. 2A, 2B).

To monitor the dynamics of hair inducing properties during the differentiation of hESCs towards DP-like cells we transplanted hESC-DPs and several related cell populations, namely, hESC differentiated for two weeks in DP medium skipping the intermediate step of NC induction (hESC), hESC-NC and partially differentiated hESC-DPs (7 days of differentiation) (Fig. 2C). Surprisingly, the transplantation of the hESC differentiated in serum conditions as well as hESC-NC resulted in significant inhibition of hair growth compared to negative control (Fig. 2C). Therefore, the differentiation of hESC-NC to hESC-DPs cells resulted in nearly 100-fold increase in hair-inducing ability (Fig. 2C). The number of hairs formed in partially differentiated hESC-NCs transplants was not significantly different than in the negative control (Fig. 2C).

These results suggest that hESC-DP cells described here have a robust hair-inducing capacity similar to that of neonatal mouse dermal cells and that hESC-NCs acquire this capacity along the differentiation procedure.

### hESC-DP incorporate into DP of de novo formed hair follicles

**Figure 3:**
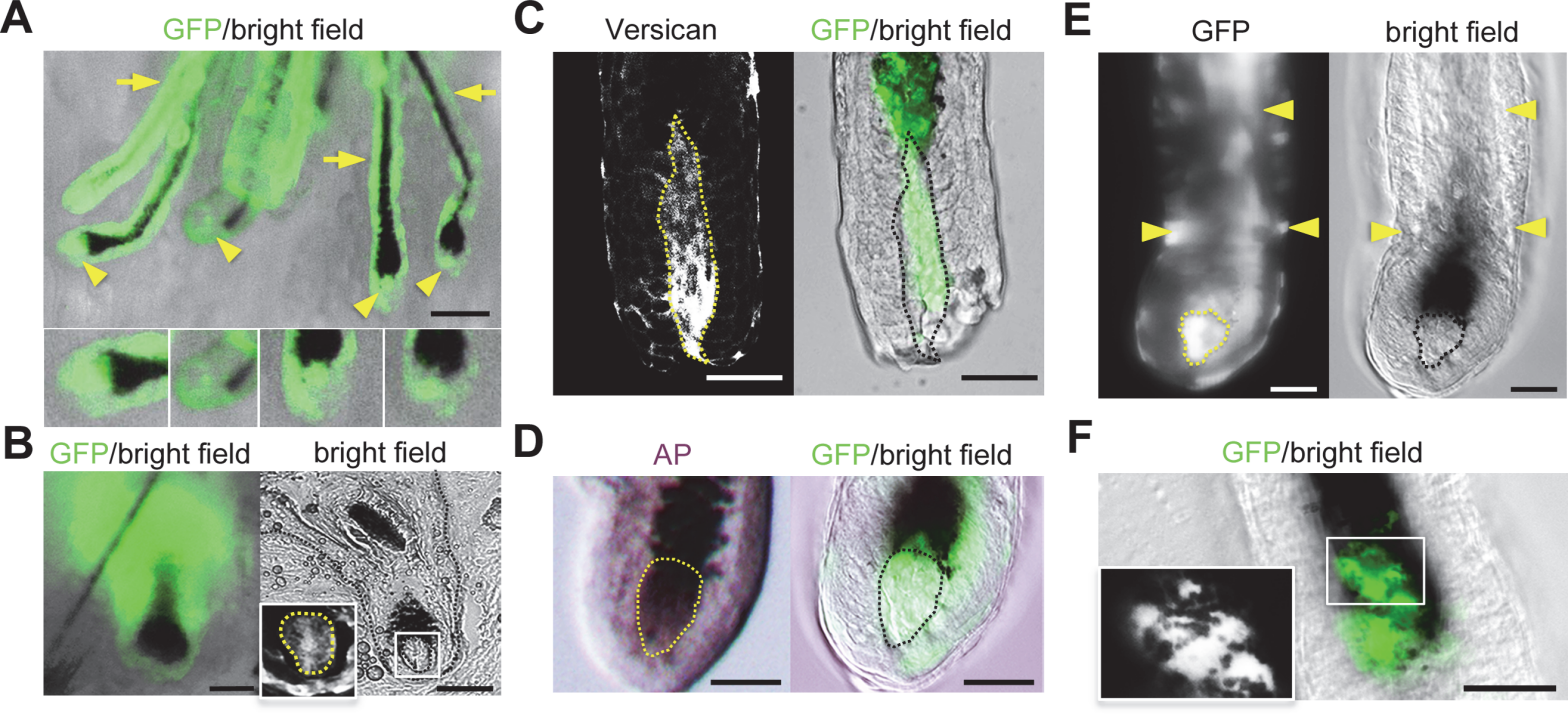
Subcutaneous transplantations of GFP-labeled hESC-DPs and hIPSC-DPs. (A) Stereoscopic observation of the whole mount transplants identified GFP-positive hESC-DP cells in positions of DP (arrows heads) and dermal capsule (arrows) in the newly formed hairs; insets show 2x enlargements of the DP regions. (B) GFP-labeled hIPSC-DPs can be found in DP and dermal capsule of the hairs: whole mount transplants (GFP/bright field) and 8um sections (bright field). Inset, fluorescence image of GFP-positive cells in the DP area of hair follicle (2x enlargement of the white square of DP area in the bright field image). (C, D) GFP-positive DPs of newly formed hairs (GFP/bright field, confocal microscopy) are positive for Versican (Versican, confocal microscopy) and Alkaline Phosphatase (AP, bright field)). (E) Rarely (~1% of newly formed hairs) NC-derived GFP-positive cells were detected in the outer root sheath area (arrows) as well as GFP-positive DP (outlined). Confocal image in the GFP panel. (F) NC-derived GFP-positive cells were found in hair matrix in transplants (confocal microscopy). Inset shows 2x enlargement of GFP-positive cells; GFP in white. Note multiple melanin granules (in black) present throughout GFP-positive cells. Scale bars 250μm for A; 50 μm for B-F.

Transplanted hESC-DP could either recruit / activate the endogenous mouse DP cells or directly mediate the observed induction of hair follicle formation. To address this question we engineered hESC-DPs to express GFP and analyzed newly formed hair follicles *in situ* (Fig. 3). Fourteen days after transplantation under the skin of nude mice using the patch method we observed a *de novo* hair formation that can be determined by the black pigmentation of the hair shafts. Stereoscopic observation of hESC-DP transplants, suggested that the majority of DPs and dermal capsules of the newly formed hairs were composed of GFP-positive cells (Fig. 3A). Confocal microscopy of the whole mount hairs isolated from hESC-DP transplants showed the presents of GFP-positive DP cells within newly induced hairs (Fig. 3C, 3D, 3E). We confirmed that GFP-positive DPs of these hairs stained positive for specific markers, namely Versican (Fig. 3C) and AP (Fig. 3D). In addition to GFP-positive DPs, we observed the presence of GFP-positive cells in the outer and the inner root sheaths area (Fig. 3E). We also observed the presence of melanin granules in the cytoplasm of GFP-positive cells in the hair matrix (Fig. 3F). Although further analysis is required to characterize the GFP-positive cells found in different compartments of hair follicles, these data suggest that transplanted hESC-DP can acquire the heir-inducing function of DP cells.

### Derivation and characterization of hIPSC-DP

In addition to H9 line of human ESC we used 3 previously characterized human iPSC lines generated from normal human BJ fibroblasts [30]. The hIPSC-NC cells were generated following previously described protocol and analyzed for the presence of neuroephitelial markers Sox2, Sox9 and nestin. We observed that hIPSC-NC obtained from all three lines showed a similar pattern of expression. Only about 50% of hIPSC-NC cells expressed Sox2 and Sox9, additionally nestin staining revealed morphological differences when compared to hESC-NC cells (S4 Fig., Fig. 1D).

hIPSC-NC cells were differentiated to obtain hIPSC-DP using the protocol described above. The immunostaining for DP markers SMA, p-75 and nestin as well as Q-PCR analysis of Versican, Nexin-1, p-75, Vimentin and SMA showed that only one IPSC line (BJ16) gave rise to cells with some expression of DP markers when compared to hESC-DP cells (S4 Fig. vs Fig. 1A, the levels of gene expression in both hIPSC-DP and hESC-DP are shown relative to hESC-NC cells).

BJ16 IPSC-DP cells were further characterized by patch transplantation. This cell population did not induce significant number of hairs when compared to negative control (data is not shown). However, the transplantation of GFP-positive BJ16 IPSC-DP cells resulted in formation of hairs with GFP-positive dermal papillae and dermal capsules albeit with much lower frequencies (1 hair out of 50) then in case of hESC-DP cells. The presence of GFP-positive cells within DP of these hairs was confirmed in sections (Fig. 3B).

Noteworthy, the integration of transplanted cells into the papillae and capsule area of newly formed hairs was observed only in the case of hESC-DP and hIPSC-DP cells. Although transplanted human DP cells engineered to express GFP were present in the dermis, these cells were never found in the DP of neighboring hair follicles (S3 Fig.). This results suggest that although human pluripotent cell-derived DP-like cells share the expression of some specific markers with DP cells isolated from adult human skin their hair-inducing capacity is higher that can enable the application of this cells for the cell-based treatment for hair loss diseases.

### Role of BMP signaling in derivation of hESC-DP

**Figure 4:**
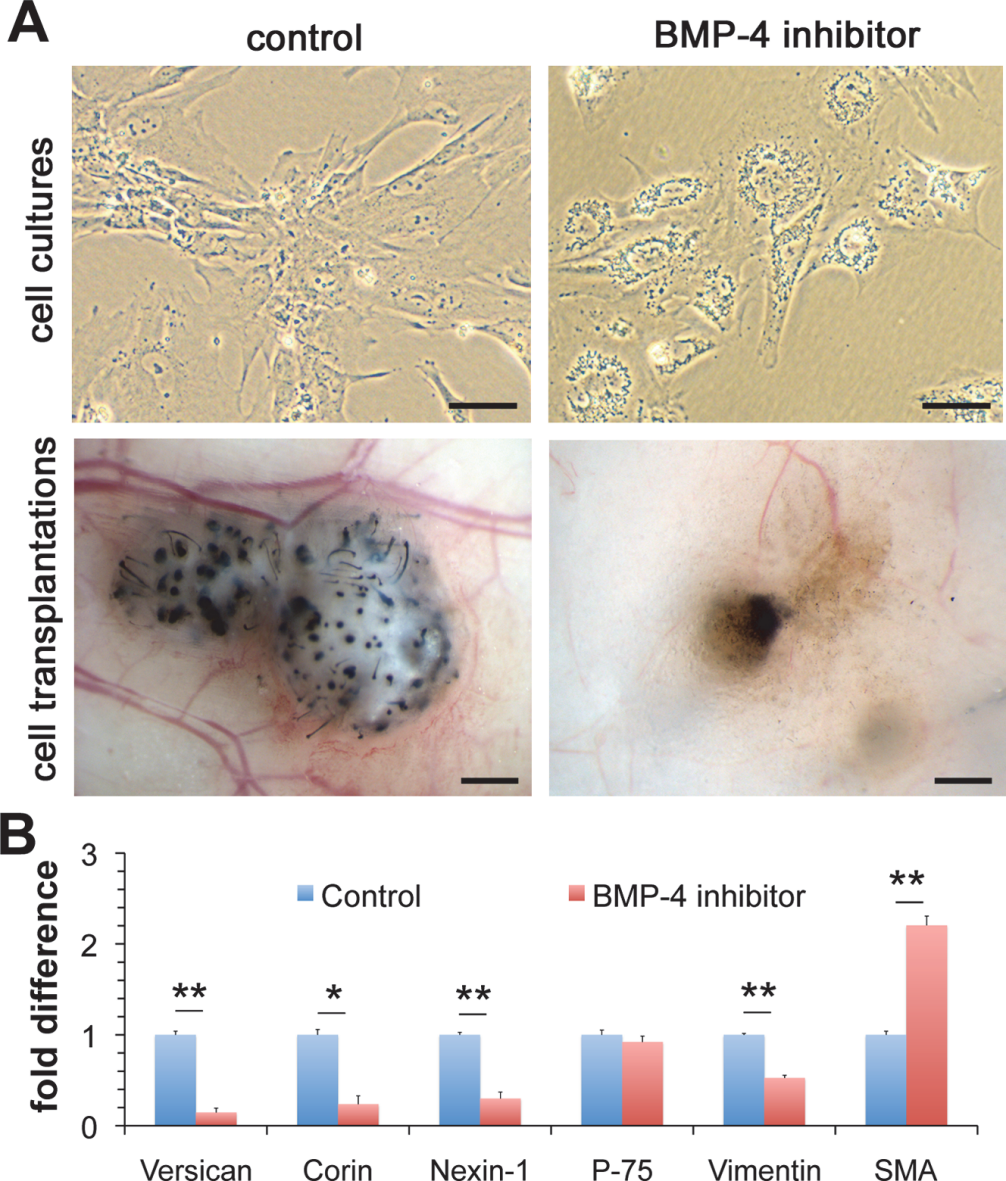
Role of BMP signaling in DP cell fate acquisition. (A) Morphology and transplantation outcomes of hESC-DP cells derived in the absence or in the presence of selective BMP inhibitor dorsomorphin. (B) Q-PCR analysis of expression of Versican, Corin, Nexin-1, p-75, Vimentin and SMA in hESC-DP cells in the absence or in the presence of selective BMP inhibitor dorsomorphin. All data are presented as mean ± SEM and were analyzed with Student’s t-test. *, P ≤ 0.05; **, P ≤ 0,001. Scale bars 50μm for cell cultures and 0.5 mm for cell transplantations.

The mechanisms involved in generation of DP from migratory NC during development are unknown. However, Fetal Bovine Serum used to induce DP differentiation from NC cells is known to contain BMPs [31], which are essential in mesoderm specification during embryo development [32,33] and mesoderm induction from hESC in vitro [34]. Critically, BMP signaling was found to be the major mechanism maintaining the hair follicle-inducing potential in mouse back skin DP [9]. We investigated whether BMP signaling plays a role in the derivation of hESC-DP cells from hESC-NC cells using a well-studied selective BMP inhibitor dorsomorphin [35]. The addition of dorsomorphin during the NC to DP conversion resulted in obvious changes in cell morphology compared to the positive control, in particular, hESC-DP cells had characteristic fibroblast-like elongated morphology whereas dorsomorphin treated cells were hexagonal and demonstrated presence on multiple granules in their cytoplasm (Fig. 4A). Furthermore, dorsomorphin treated cells transplanted under the Nude mice skin were unable to induce hair formation suggesting the loss of DP properties (Fig. 4A). Q-PCR analysis of dorsomorphin treated cells revealed that the inhibition of BMP signaling resulted in significant decrease in levels of mRNAs encoding the DP-specific markers Versican (P = 0.0002), Corin (P = 0.0022), Nexin-1 (P = 0.0008) and Vimentin (P = 0.0001) but not the pan NC marker p-75 (not changed) or the smooth muscle marker SMA (significantly increased) (Fig. 4B). However, we were not able to derive DP cells from hESC-NC cultures using BMPs as the only differentiation agents (data not shown) suggesting other signaling pathways to be involved in cephalic DP cell fate acquisition. These results indicate that BMP singling is necessary but not sufficient for the generation and/or maintenance of hair-inducing hESC-DP from hESC-NC cells.

## Discussion

The results suggest that hESC-derived NC cells cultured in serum containing medium progressively acquire the markers of human DP cells and give rise to adherent cell population with hair-inducing potential. We speculate that robust hair-inducing capacity of hESC-DP as compared to human DP cells might reflect a major resemblance of the former to an embryonic or neonatal population of dermal papilla precursor cells, which are known to induce hair follicle formation through different mechanisms [36].

The hESC-DP cultures are likely to be heterogeneous. The average hair inducing capacity shown by these cultures was similar to that of neonatal mouse dermal cells. Prospectively purified primary mouse DP cells were shown to be ~5 times more efficient in hair induction than mouse dermal preparations [27]. Therefore, it is possible that hESC-DP cultures comprise a sub-population of hair inducing DP-like cells, with even higher heir-inducing capacity than the average reported above. Additionally, when GFP-positive hESC-DP were transplanted we observed the presence of GFP-positive cells in other compartments of hair follicles (i.e. outer root sheath, inner root sheath and hair matrix). Since melanocytes are known to originate from migratory NC during development, it is likely that some hESC-NC cells give rise to melanocyte precursors within hESC-DP cultures. Similarly, it is possible that some hESC-NCs are able to give rise to keratinocytes of newly formed hairs since rodent NC cells can give rise to the epidermal stem cells in the whisker’s bulge [37]. These data suggest that hESC-DP is a mixed population of NC-derived cells that contain DP-like cells with hair-inducing properties, but also might contain melanocyte and keratinocyte forming cells. Further analysis of hESC-DP cultures is needed to address this question.

Our results suggest that the intermediate step of hESC differentiation into the NC lineage seems is critical, skipping the NC induction results in a complete loss of hair-inducing activity. We speculate that directing hESCs to the NC cells might limit the variety of mesenchymal cell types to the subset that is developmentally specified downstream from NC cells in skin (e.g. cephalic DP during development, melanocytes, cephalic bulge). Therefore, the hESC-DP-like cells become prominently enriched in heir-inducing DP-like cells using relatively common mesenchymal-enriching conditions such as differentiation in serum containing medium and selection for the adherent cell types.

Different iPSC lines may have variable propensity to differentiate towards DP-like cells. Indeed, we had only modest success in generating DP-like cells with 1 out of 3 hiPSC lines used. The hiPSC-DP cells were not able to induce hair follicle formation when transplanted using patch method and had low frequency of incorporation into the DP of newly formed hair follicles. We speculate that this might be a result of the epigenetic memory phenomenon, known to influence IPSC differentiation [38,39,40]. The IPSC lines used for our experiments were derived from BJ fibroblasts [30]. Their mesodermal origin could cause difficulties on the first step of differentiation—induction of the ectodermal neural crest cells. Indeed, only some hIPSC-NC cells expressed neuroepithelial markers Sox2 and Sox9 (S4 Fig.). However, a global comparison of multiple hiPCS and hESC lines suggested that when sufficiently large numbers of hiPSC lines were compared with hESC lines a major overlap in their differentiation potential was observe [41]. Therefore although the absolute efficiency may vary between different hESC and hiPSC lines it should be possible to derive cells with hair-inducing properties from many hiPSC [42].

Recently, SKPs were shown to be highly potent in hair induction [27], but progressive loss of SKPs in a process of aging might hamper the isolation of autologous SKPs for hair regenerative therapies for aged people [43]. The derivation of hair-inducing DP-like cells from hESCs represents the first step towards development of a cell-based treatment for people with hair loss diseases.

## Materials and Methods

### hESCs culture

H9 hESC line was maintained on eradiated mouse embryonic fibroblast feeder layers as previously described in details [24].

### Generation of NC cells from hESCs

The differentiation protocol was previously described in details [24,25]. Briefly, neurospheres were generated by manual separation of hESCs colonies, growing them in low adhesion dishes (Ted Pella, Redding, CA) as floating spheres for five days. On day 5 neurospheres were plated on fibronectin- (1 mg/ml) or matrigel-coated culture flasks and maintained with Neutralization Medium [24].

### Generation of ESC-DP

NC cells on passage one were cultured for 3 days with DP Medium of the following composition: DMEM/F-12 Glutamax (Gibco 10829-018), 10% FBS (Gibco #10437), 1mM L-glutamine (Gibco 25030-081), 1X antibiotic/antimycotic (Omega Scientific AA-40). After that, they were dissociated to single-cell suspension with 0.25% Trypsin-EDTA solution (Gibco 25200) and plated on uncoated culture dishes in density 100 thousands cells per square sm. Floating cells failed to attach after 24 hours were removed with medium change on the following day. Attached cells were grown on uncoated plastic dishes with Medium change every other day; culture was passed every 4–5 days.

### Immunohistochemistry and FACS analysis

Cell cultures were fixed with 4% PFA in PBS for 10 minutes at room temperature; tissue samples were fixed at 4° overnight. After fixation cells were washed in PBS 3 times for 5 minutes, and tissue samples were embedded in OCT for frozen sections or used for hair dissections and whole mount preparations. Cells and sections were blocked in 4% BSA or 10% goat serum with 0.05%- 0.3% Triton X 100 (Sigma T8787) in PBS for one hour prior to staining. Primary antibodies were applied over night at 4°C. Cells or sections were then washed in PBS for 3 times 15 minutes each. Secondary antibodies (diluted 1:500 in PBS) were applied for 1 hour at room temperature in dark. Cells were washed in PBS for 3 times 10 minutes each. Nuclei were labeled with Hoechst or Dapi. For FACS analysis, cells of interest were detached with Accutase and resuspended in 3% BSA/PBS for 20 minutes to block non-specific binding. Then cells were incubated with primary antibodies for 30 minutes on ice. Then cells were washed in 3 ml of PBS and resuspended in 3% BSA/PBS with appropriate secondary antibodies (1:1000) for 30 minutes and washed with 3 mls of PBS before being resuspended in 1% BSA/PBS and incubated with PI (live/dead stain). Cells were sorted according to fluorescence (FACSVantageSE DiVa, BD Biosciences, San Jose) and data were analyzed with FlowJo software.

The following antibodies were used: mouse monoclonal CD47(R&D), mouse monoclonal CD184 (eBioscience), mouse monoclonal CD44 (Novus), goat polyclonal Foxd3 (Santa Cruz), rabbit polyclonal GFP (Invitrogen), mouse monoclonal ITGA4 (R&D), mouse monoclonal Nestin (Chemicon), mouse monoclonal OCT3/4 (Santa Cruz), rabbit polyclonal P-75 (Chemicon), mouse monoclonal SMA (Chemicon), rabbit polyclonal Sox2 (Abcam), rabbit polyclonal Sox9 (Millipore), rabbit polyclonal Sox10 (Abcam), mouse monoclonal SSEA4 (R&D), mouse monoclonal Versican (Seikagaku Corporation).

### Q-PCR

Total RNA was extracted using the RNeasy kit and 1 μg of total RNA was reverse transcribed using the Quantitect kit (Qiagen) according to the manufacturer's suggestions to make cDNA. Q-PCR was performed with SyberGreen master mix (Invitrogen) according to the manufacturer's recommendation. For Q-PCR, 18S expression level was used for normalization and the data were analyzed using the standard curve method. Q-PCR was perfomed as follows: Initial denaturation: 10 min at 95°C; 40 cycles of denaturation: 30 sec at 95°C, annealing: 1 min at 56°C, extension: 30 sec at 72°C; and one final cycle of denaturation for 1 min at 95°C, annealing for 30 sec at 65°C and final denaturation for 30 sec at 95°C. Primers used summarized in Table 1.

**Table 1:** Real time Q-PCR primers.

Name	Forward primer	Reversed primer
18S	AGTCCCTGCCCTTTGTACACA	CGATCCGAGGGCCTCACTA
Corin	AACCCAGTGGACATATCTGTGGCT	TGTTGATGCCAAGCACCACTTTCC
Nexin	TGTGAAGTCGAGGCCTCATGACAA	TCTTGGAGACGATGGCCTTGTTGA
P-75	TTCAAGGGCTTACACGTGGAGGAA	AATTCCTTCTTGCCGCATTCCCAC
Versican	TGAGCATGACTTCCGTTGGACTGA	CCACTGGCCATTCTCATGCCAAAT
Vimentin	AGAACCTGCAGGAGGCAGAAGAAT	TTCCATTTCACGCATCTGGCGTTC

### Cells: ESC-DP cells on different passages were obtained according to described protocol

Cells were dissociated to single-cell suspension with 0.1% Trypsin-EDTA (Gibco 25200) and washed 3 times with PBS by serial centrifuging (1200 RPM for 5 minutes). Finally cells were resuspended in DP medium at concentration 5 million per 100 μl and kept on ice until transplanted.

### Mouse epidermal and dermal cells were obtained from p0-p2 BL6 mice skin

Back skins were isolated and placed on ice, washed in PBS with antibiotic/antimycotic (final 1X; Omega Scientific AA-40) 5 times for 5 minutes shaking and incubated in 0.01% Dispase (Sigma) in PBS overnight at 40C. Next day epidermal layers of skins were isolated with forceps, washed in PBS for 5 min and incubated in 0.1% Trypsin-EDTA solution at 370 C for 8 minutes. Dermal layers of skin were homogenized with scissors and digested with 0.1% Trypsin-EDTA solution at 370 C for 45 minutes. Enzyme activity was blocked with addition of DP medium and epidermises or dermises were pipetted vigorously for 10 minutes. Cell suspension was isolated with cell strainer (BD Falcon 9261365) and washed in PBS by centrifuging 3 times (1200 RPM for 5 minutes). Cells were resuspended in DP medium at 5 million cells per 100 μl and kept on ice until transplanted. All procedures were performed after Burnham Institute Animal Committee approval and following the NIH guidelines for working with animals.

### Human dermal papillae cells

The use of human tissue-derived samples in this study was limited to use of skin waste tissue from 2 cosmetic medical procedures. The Sanford-Burnham IRB review determined that written informed consent for this research was provided by the subjects. Skins were washed in PBS with antibiotic/antimycotic (final 1X; Omega Scientific AA-40) 15 times for 5 minutes shaking. Then fat containing hair follicles was isolated with scalpel and incubated in 0.2% Dispase (Sigma) in DMEM/F12 overnight at 40C. Next day hair follicles were isolated from fat with the forceps and incubated in 0.1% Collagenase type I (Sigma) in DMEM/F12 for 5–7 hours. Then DPs were detached from the rest of follicles by vigorous pipetting for 10 minutes. After hair follicles settle on the bottom DP staid in supernatant and were isolated by centrifuging 1000 RPM for 5 minutes.

### Cells transplantation

The method of cell transplantation was described previously in detail [27]. Briefly, 100 μl of suspension (5x105 cells) of cells of interest (ESC-NC, early ESC-DP, ESC-DP, rDP p2) were combined with 100 μl of suspension of epidermal cells (5x105 cells) and transplanted with subcutaneous injections to immunodeficient mice (strain Athymic Nu/Nu). After 14–21 days mice were euthanized and transplants were analyzed. For negative control epidermal cells alone were transplanted.

## Supporting Information

S1 Fig Expression of mesenchymal markers in hESC-NC. Flow cytometry analysis of mesenchymal markers (CD47, CD184, CD44) expression in hESC-NC cultures.
(TIF)

S2 Fig Expression of DP markers in human hair follicles DP cells *in situ*. Immunofluorescent staining for SMA, Alkaline Phosphatase (AP), Nestin and Versican Frozen sections; DAPI in blue. Scale bars 100 μm.
(TIF)

S3 Fig Transplantation of human DP cells under the skin of Nude mice. GFP-positive hDP cells can be found in dermis but do not incorporate into the DP areas of the newly formed hairs; whole mount transplant (insets shows 2x enlargements of the DP areas). Scale bar 250 μm.
(TIF)

S4 Fig Differentiation of hIPSCs into DP-like cells via NC intermediate. (A) Expression of neuroephitelial markers Sox 2, Sox 9 and nestin in hIPSC-NC cultures. Immunofluorescent staining, DAPI in blue. (B) Expression of DP markers Smooth Muscle Actin (SMA), P-75 and Nestin in human IPSC-DP cell cultures. Immunofluorescent staining, DAPI in blue. (C) Q-PCR analysis of expression of Versican, Nexin-1, p-75, Vimentin and SMA. The levels of gene expression normalized to 18S and shown as log fold change over hESC-NC level of expression. Scale bars 100 μm.
(TIF)

## References

[1] Hardy MH (1992) The secret life of the hair follicle. Trends Genet 8: 55–61. 1566372 10.1016/0168-9525(92)90350-d

[2] Jahoda CA, Reynolds AJ (1996) Dermal-epidermal interactions. Adult follicle-derived cell populations and hair growth. Dermatol Clin 14: 573–583. 9238317 10.1016/s0733-8635(05)70385-5

[3] Millar SE (2002) Molecular mechanisms regulating hair follicle development. J Invest Dermatol 118: 216–225. 11841536 10.1046/j.0022-202x.2001.01670.x

[4] Driskell RR, Clavel C, Rendl M, Watt FM (2011) Hair follicle dermal papilla cells at a glance. J Cell Sci 124: 1179–1182. 10.1242/jcs.082446 21444748 PMC3115771

[5] Schmidt-Ullrich R, Paus R (2005) Molecular principles of hair follicle induction and morphogenesis. Bioessays 27: 247–261. 15714560 10.1002/bies.20184

[6] Botchkarev VA, Paus R (2003) Molecular biology of hair morphogenesis: development and cycling. J Exp Zool B Mol Dev Evol 298: 164–180. 12949776 10.1002/jez.b.33

[7] Rendl M, Lewis L, Fuchs E (2005) Molecular dissection of mesenchymal-epithelial interactions in the hair follicle. PLoS Biol 3: e331 16162033 10.1371/journal.pbio.0030331 PMC1216328

[8] Kishimoto J, Burgeson RE, Morgan BA (2000) Wnt signaling maintains the hair-inducing activity of the dermal papilla. Genes Dev 14: 1181–1185. 10817753 PMC316619

[9] Rendl M, Polak L, Fuchs E (2008) BMP signaling in dermal papilla cells is required for their hair follicle-inductive properties. Genes Dev 22: 543–557. 10.1101/gad.1614408 18281466 PMC2238674

[10] Greco V, Chen T, Rendl M, Schober M, Pasolli HA, et al (2009) A two-step mechanism for stem cell activation during hair regeneration. Cell Stem Cell 4: 155–169. 10.1016/j.stem.2008.12.009 19200804 PMC2668200

[11] Weinberg WC, Goodman LV, George C, Morgan DL, Ledbetter S, et al (1993) Reconstitution of hair follicle development in vivo: determination of follicle formation, hair growth, and hair quality by dermal cells. J Invest Dermatol 100: 229–236. 8440892 10.1111/1523-1747.ep12468971

[12] Driskell RR, Giangreco A, Jensen KB, Mulder KW, Watt FM (2009) Sox2-positive dermal papilla cells specify hair follicle type in mammalian epidermis. Development 136: 2815–2823. 10.1242/dev.038620 19605494 PMC2730408

[13] Jahoda CA, Horne KA, Oliver RF (1984) Induction of hair growth by implantation of cultured dermal papilla cells. Nature 311: 560–562. 6482967 10.1038/311560a0

[14] Jahoda CA, Oliver RF, Reynolds AJ, Forrester JC, Gillespie JW, et al (2001) Trans-species hair growth induction by human hair follicle dermal papillae. Exp Dermatol 10: 229–237. 11493311 10.1034/j.1600-0625.2001.100402.x

[15] Wu JJ, Zhu TY, Lu YG, Liu RQ, Mai Y, et al (2006) Hair follicle reformation induced by dermal papilla cells from human scalp skin. Arch Dermatol Res 298: 183–190. 16897077 10.1007/s00403-006-0686-9

[16] Chermnykh ES, Vorotelyak EA, Gnedeva KY, Moldaver MV, Yegorov YE, et al (2010) Dermal papilla cells induce keratinocyte tubulogenesis in culture. Histochem Cell Biol 133: 567–576. 10.1007/s00418-010-0691-0 20336308

[17] Lichti U, Weinberg WC, Goodman L, Ledbetter S, Dooley T, et al (1993) In vivo regulation of murine hair growth: insights from grafting defined cell populations onto nude mice. J Invest Dermatol 101: 124S–129S. 8326145 10.1111/1523-1747.ep12363165

[18] Yang CC, Cotsarelis G (2010) Review of hair follicle dermal cells. J Dermatol Sci 57: 2–11. 10.1016/j.jdermsci.2009.11.005 20022473 PMC2818774

[19] Bronner-Fraser M (1994) Neural crest cell formation and migration in the developing embryo. FASEB J 8: 699–706. 8050668 10.1096/fasebj.8.10.8050668

[20] Danielian PS, Muccino D, Rowitch DH, Michael SK, McMahon AP (1998) Modification of gene activity in mouse embryos in utero by a tamoxifen-inducible form of Cre recombinase. Curr Biol 8: 1323–1326. 9843687 10.1016/s0960-9822(07)00562-3

[21] Fernandes KJ, McKenzie IA, Mill P, Smith KM, Akhavan M, et al (2004) A dermal niche for multipotent adult skin-derived precursor cells. Nat Cell Biol 6: 1082–1093. 15517002 10.1038/ncb1181

[22] Nagoshi N, Shibata S, Kubota Y, Nakamura M, Nagai Y, et al (2008) Ontogeny and multipotency of neural crest-derived stem cells in mouse bone marrow, dorsal root ganglia, and whisker pad. Cell Stem Cell 2: 392–403. 10.1016/j.stem.2008.03.005 18397758

[23] Metallo CM, Ji L, de Pablo JJ, Palecek SP (2008) Retinoic acid and bone morphogenetic protein signaling synergize to efficiently direct epithelial differentiation of human embryonic stem cells. Stem Cells 26: 372–380. 17962700 10.1634/stemcells.2007-0501

[24] Curchoe CL, Maurer J, McKeown SJ, Cattarossi G, Cimadamore F, et al (2010) Early acquisition of neural crest competence during hESCs neuralization. PLoS One 5: e13890 10.1371/journal.pone.0013890 21085480 PMC2976694

[25] Cimadamore F, Fishwick K, Giusto E, Gnedeva K, Cattarossi G, et al (2011) Human ESC-Derived Neural Crest Model Reveals a Key Role for SOX2 in Sensory Neurogenesis. Cell Stem Cell 8: 538–551. 10.1016/j.stem.2011.03.011 21549328 PMC4110917

[26] Mould AP, Askari JA, Craig SE, Garratt AN, Clements J, et al (1994) Integrin alpha 4 beta 1-mediated melanoma cell adhesion and migration on vascular cell adhesion molecule-1 (VCAM-1) and the alternatively spliced IIICS region of fibronectin. J Biol Chem 269: 27224–27230. 7525548

[27] Biernaskie J, Paris M, Morozova O, Fagan BM, Marra M, et al (2009) SKPs derive from hair follicle precursors and exhibit properties of adult dermal stem cells. Cell Stem Cell 5: 610–623. 10.1016/j.stem.2009.10.019 19951689 PMC2828150

[28] Hoogduijn MJ, Gorjup E, Genever PG (2006) Comparative characterization of hair follicle dermal stem cells and bone marrow mesenchymal stem cells. Stem Cells Dev 15: 49–60. 16522162 10.1089/scd.2006.15.49

[29] Pittenger MF, Mackay AM, Beck SC, Jaiswal RK, Douglas R, et al (1999) Multilineage potential of adult human mesenchymal stem cells. Science 284: 143–147. 10102814 10.1126/science.284.5411.143

[30] Liu GH, Barkho BZ, Ruiz S, Diep D, Qu J, et al. (2011) Recapitulation of premature ageing with iPSCs from Hutchinson-Gilford progeria syndrome. Nature. 10.1038/nature09879 PMC3088088 21346760

[31] Kodaira K, Imada M, Goto M, Tomoyasu A, Fukuda T, et al (2006) Purification and identification of a BMP-like factor from bovine serum. Biochem Biophys Res Commun 345: 1224–1231. 16716261 10.1016/j.bbrc.2006.05.045

[32] Winnier G, Blessing M, Labosky PA, Hogan BL (1995) Bone morphogenetic protein-4 is required for mesoderm formation and patterning in the mouse. Genes Dev 9: 2105–2116. 7657163 10.1101/gad.9.17.2105

[33] Mishina Y, Suzuki A, Ueno N, Behringer RR (1995) Bmpr encodes a type I bone morphogenetic protein receptor that is essential for gastrulation during mouse embryogenesis. Genes Dev 9: 3027–3037. 8543149 10.1101/gad.9.24.3027

[34] Zhang P, Li J, Tan Z, Wang C, Liu T, et al (2008) Short-term BMP-4 treatment initiates mesoderm induction in human embryonic stem cells. Blood 111: 1933–1941. 18042803 10.1182/blood-2007-02-074120

[35] Yu PB, Hong CC, Sachidanandan C, Babitt JL, Deng DY, et al (2008) Dorsomorphin inhibits BMP signals required for embryogenesis and iron metabolism. Nat Chem Biol 4: 33–41. 18026094 10.1038/nchembio.2007.54 PMC2727650

[36] Inamatsu M, Tochio T, Makabe A, Endo T, Oomizu S, et al (2006) Embryonic dermal condensation and adult dermal papilla induce hair follicles in adult glabrous epidermis through different mechanisms. Dev Growth Differ 48: 73–86. 16512852 10.1111/j.1440-169X.2006.00848.x

[37] Sieber-Blum M, Grim M (2004) The adult hair follicle: cradle for pluripotent neural crest stem cells. Birth Defects Res C Embryo Today 72: 162–172. 15269890 10.1002/bdrc.20008

[38] Kim K, Doi A, Wen B, Ng K, Zhao R, et al (2010) Epigenetic memory in induced pluripotent stem cells. Nature 467: 285–290. 10.1038/nature09342 20644535 PMC3150836

[39] Jandial R, Levy ML (2011) Cellular alchemy: induced pluripotent stem cells retain epigenetic memory. World Neurosurg 75: 5–6. 10.1016/j.wneu.2011.01.013 21492641

[40] Bar-Nur O, Russ HA, Efrat S, Benvenisty N (2011) Epigenetic memory and preferential lineage-specific differentiation in induced pluripotent stem cells derived from human pancreatic islet Beta cells. Cell Stem Cell 9: 17–23. 10.1016/j.stem.2011.06.007 21726830

[41] Boulting GL, Kiskinis E, Croft GF, Amoroso MW, Oakley DH, et al (2011) A functionally characterized test set of human induced pluripotent stem cells. Nat Biotechnol 29: 279–286. 10.1038/nbt.1783 21293464 PMC3229307

[42] Bock C, Kiskinis E, Verstappen G, Gu H, Boulting G, et al (2011) Reference Maps of human ES and iPS cell variation enable high-throughput characterization of pluripotent cell lines. Cell 144: 439–452. 10.1016/j.cell.2010.12.032 21295703 PMC3063454

[43] Zouboulis CC, Adjaye J, Akamatsu H, Moe-Behrens G, Niemann C (2008) Human skin stem cells and the ageing process. Exp Gerontol 43: 986–997. 10.1016/j.exger.2008.09.001 18809487

