# Supplementary figures and images for: Derivation of Hair-Inducing Cell from Human Pluripotent Stem Cells
Source: PLoS One. 2015 Jan 21;10(1):e0116892. doi: 10.1371/journal.pone.0116892 (PMC4301813; doi:10.1371/journal.pone.0116892)

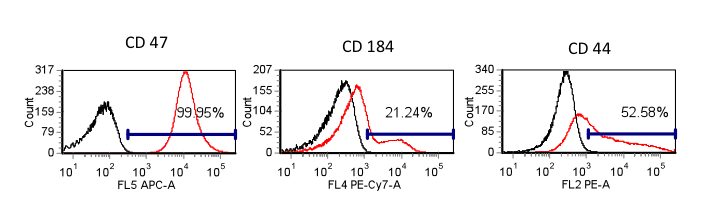

Supplement: S1 Fig — Flow cytometry analysis of mesenchymal markers (CD47, CD184, CD44) expression in hESC-NC cultures. (TIF) [file pone.0116892.s001.tif]

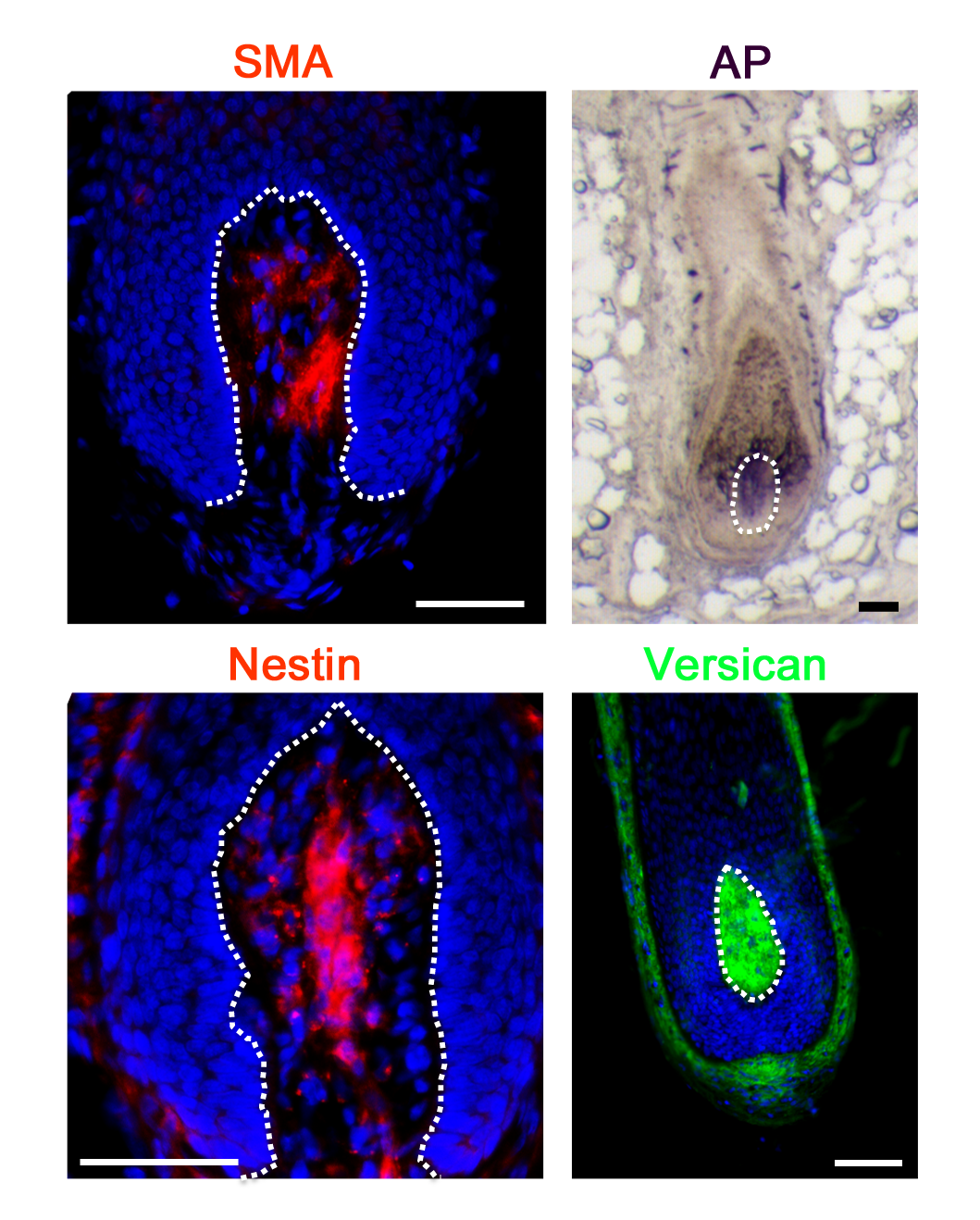

Supplement: S2 Fig — Immunofluorescent staining for SMA, Alkaline Phosphatase (AP), Nestin and Versican Frozen sections; DAPI in blue. Scale bars 100 μm. (TIF) [file pone.0116892.s002.tif]

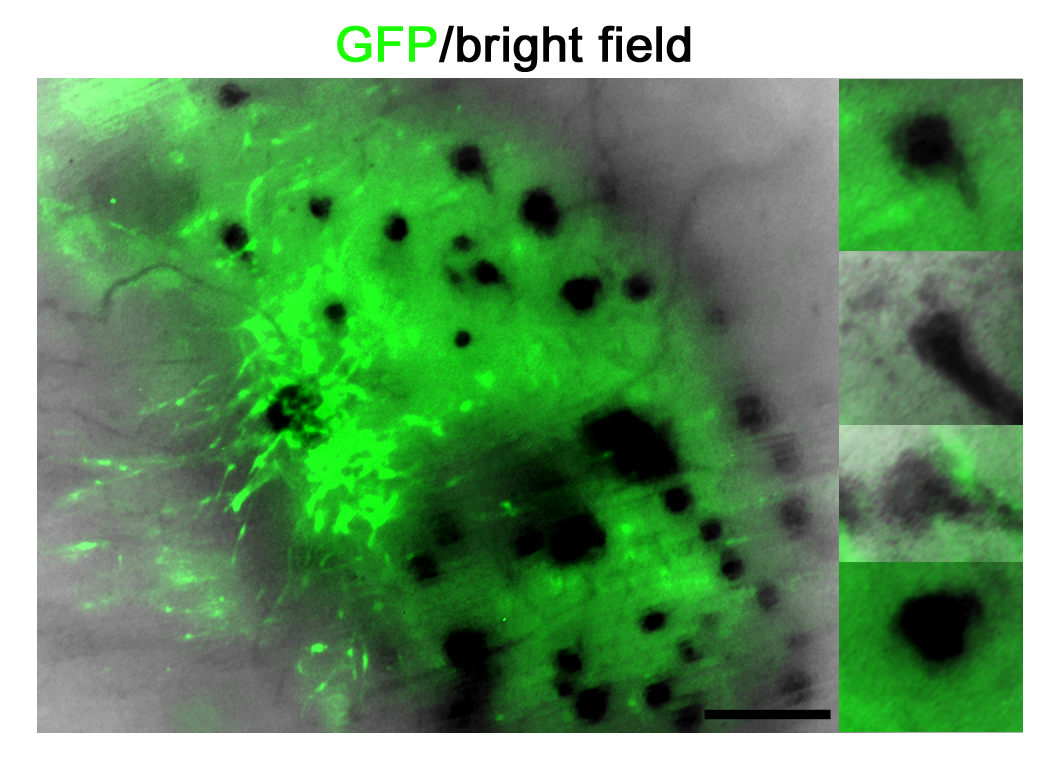

Supplement: S3 Fig — GFP-positive hDP cells can be found in dermis but do not incorporate into the DP areas of the newly formed hairs; whole mount transplant (insets shows 2x enlargements of the DP areas). Scale bar 250 μm. (TIF) [file pone.0116892.s003.tif]

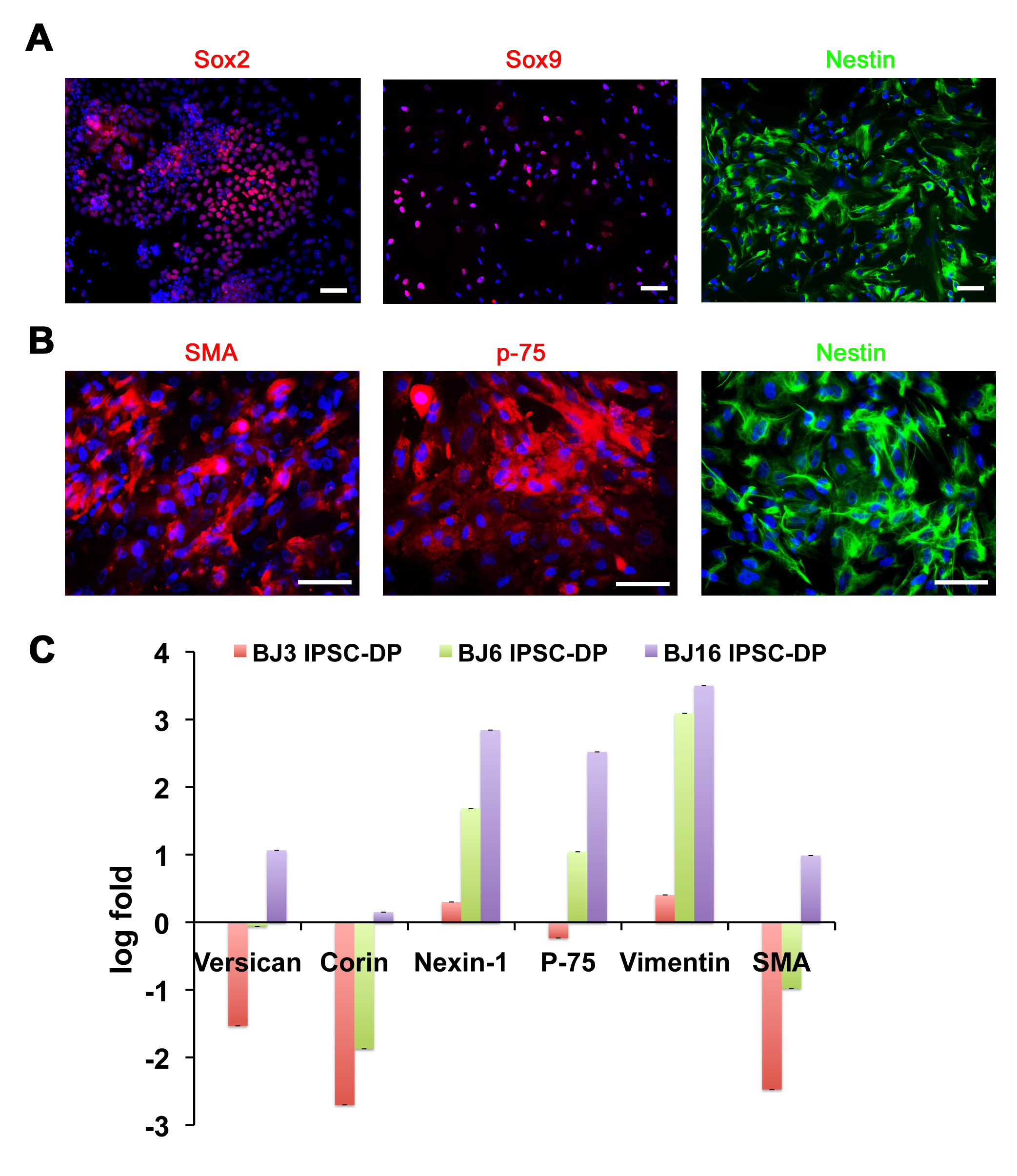

Supplement: S4 Fig — (A) Expression of neuroephitelial markers Sox 2, Sox 9 and nestin in hIPSC-NC cultures. Immunofluorescent staining, DAPI in blue. (B) Expression of DP markers Smooth Muscle Actin (SMA), P-75 and Nestin in human IPSC-DP cell cultures. Immunofluorescent staining, DAPI in blue. (C) Q-PCR analysis of expression of Versican, Nexin-1, p-75, Vimentin and SMA. The levels of gene expression normalized to 18S and shown as log fold change over hESC-NC level of expression. Scale bars 100 μm. (TIF) [file pone.0116892.s004.tif]
